# Association of Diphtheria-Tetanus–Acellular Pertussis Vaccine Timeliness and Number of Doses With Age-Specific Pertussis Risk in Infants and Young Children

**DOI:** 10.1001/jamanetworkopen.2021.19118

**Published:** 2021-08-10

**Authors:** Madhura S. Rane, Pejman Rohani, M. Elizabeth Halloran

**Affiliations:** 1 Department of Epidemiology, University of Washington, Seattle; 2 Institute for Implementation Science in Population Health, City University of New York, New York; 3 Odum School of Ecology, University of Georgia, Athens; 4 Department of Infectious Diseases, University of Georgia, Athens; 5 Fred Hutchinson Cancer Research Center, Seattle, Washington; 6 Department of Biostatistics, University of Washington, Seattle

## Abstract

**Importance:**

In most countries, the diphtheria-tetanus–acellular pertussis (DTaP) vaccine is administered as a 3-dose infant series followed by additional booster doses in the first 5 years of life. Short-term immunity from the DTaP vaccine can depend on the number, timing, and interval between doses. Not receiving doses in a timely manner might be associated with a higher pertussis risk.

**Objective:**

To examine the association between number and timeliness of vaccine doses and age-specific pertussis risk.

**Design, Setting, and Participants:**

This population-based, retrospective cohort study used Washington State Immunization Information System data and pertussis surveillance data from Public Health Seattle and King County, Washington. Included participants were children aged 3 months to 9 years born or living in King County, Washington, between January 1, 2008, and December 31, 2017. Data were analyzed from June 30 to December 1, 2019.

**Exposures:**

Being undervaccinated (receiving fewer than recommended doses at a given age) or delayed vaccination (not receiving doses within time frames recommended by Centers for Disease Control and Prevention).

**Main Outcomes and Measures:**

Suspected, probable, and confirmed pertussis diagnosis.

**Results:**

A total of 316 404 children (median age, 65.2 months [interquartile range, 35.3-94.1 months]; 162 025 boys [51.2%]) as of December 31, 2017, with 17.4 million person-months of follow-up were included in the analysis. A total of 19 943 children (6.3%) had no vaccines recorded in the Immunization Information System, 116 193 (36.7%) received a vaccine with a delay, and 180 268 (56.9%) were fully vaccinated with no delay. Delayed vaccination and undervaccination rates were higher for older children (17.6% delayed or undervaccinated at age 2 months for dose 1 at 3 months vs 41.6% at age 5 years for dose 5) but improved for successive birth cohorts (52.2% for 2008 birth cohort vs 32.3% for 2017 birth cohort). Undervaccination was significantly associated with higher risk of pertussis for the 3-dose primary series (adjusted relative risk [aRR], 4.8; 95% CI, 3.1-7.6), the first booster (aRR, 3.2; 95% CI, 2.3-4.5), and the second booster (aRR, 4.6; 95% CI, 2.6-8.2). However, delay in vaccination among children who received the recommended number of vaccine doses was not associated with pertussis risk.

**Conclusions and Relevance:**

The results of this cohort study suggest that undervaccination is associated with higher pertussis risk. Short delays in vaccine receipt may be less important if the age-appropriate number of doses is administered, but delaying doses is not recommended. Ensuring that children receive all doses of pertussis vaccine, even if there is some delay, is important.

## Key Points

**Question:**

Are undervaccination and delay in vaccination with diphtheria-tetanus–acellular pertussis (DTaP) vaccine associated with pertussis risk in infants and young children?

**Findings:**

In this population-based cohort study of 316 404 children, those who were undervaccinated for the primary series, second-year booster, and preschool booster of the DTaP vaccine had 4.8-fold, 3.2-fold, and 4.6-fold higher pertussis risk, respectively, compared with fully vaccinated children. Doses administered with a short delay were not associated with pertussis risk.

**Meaning:**

These results suggest that young children should receive DTaP doses in an age-appropriate and timely fashion as recommended by the Centers for Disease Control and Prevention for best protection against childhood pertussis.

## Teaser

This cohort study assesses whether undervaccination or a delay in vaccination with the diphtheria-tetanus–acellular pertussis (DTaP) vaccine is associated with pertussis risk in infants and young children.

## Introduction

Widespread rollout of the diphtheria-tetanus–whole-cell pertussis (DTwP) vaccines in the 1940s resulted in a dramatic decrease in pediatric pertussis incidence until the 1970s and 1980s in the US.^1,2^ Owing to concerns surrounding the safety and reactogenicity of DTwP vaccines, a less reactogenic diphtheria-tetanus–acellular pertussis (DTaP) vaccine was developed.^1,3^ The DTaP vaccines are safe and efficacious, and most developed countries recommend them for their infant primary series in national immunization programs.^4^ Preschool and adolescent booster doses are also included in immunization schedules, especially in high-income countries, owing to concerns about increases in age at infection and waning vaccine-induced immunity.^5^ Despite high vaccination coverage for both the primary series and boosters, the US has experienced a resurgence in pertussis since the 1990s.^6^

Waning of DTaP-induced immunity has been widely cited as one of the main drivers of pertussis resurgence in countries with high vaccination coverage.^7,8,9,10^ Short-term protection afforded by pertussis vaccines depends on the number, timing, and interval between doses.^11^ Thus, strategic scheduling and timely uptake of boosters is crucial. Longer intervals between doses due to delays or missed immunizations could increase pertussis risk even in partially vaccinated children. This increased risk could lead to sustained transmission of pertussis and periodic outbreaks.^12,13^ Observational studies in the US and Taiwan have suggested that undervaccination or delay in vaccination results in higher pertussis risk.^14,15,16,17^ Current methods of estimating DTaP vaccination coverage at specific ages without estimating timeliness of each dose can mask delays in vaccination while showing high vaccination coverage at the national level.^12,13,18^

Our objective was to examine the association between number and timeliness of vaccine doses and age-specific pertussis risk in infants and young children registered in the Washington Immunization Information System (WA-IIS). Using log-binomial models, we compared pertussis risk between children who were age-appropriately vaccinated and those who were undervaccinated or had delayed vaccination.

## Methods

### Pertussis Cases

Pertussis cases reported between January 1, 2008, and December 31, 2017, in children aged 3 months to 9 years were obtained from the Public Health Seattle and King County (PHSKC) surveillance database. The clinical case definition of pertussis used was a cough illness lasting 2 weeks or more with at least 1 of the following: paroxysms of coughing or inspiratory “whoop,” posttussive vomiting, or apnea (with or without cyanosis) for infants up to 1 year of age. Suspected, probable, and confirmed pertussis cases were included.^19^ Pertussis remains highly underreported,^20,21^ and only cases of patients who were symptomatic and whose caregivers sought medical help through PHSKC were included. Information on age, sex, and home address for patients was available. We geocoded the home addresses of patients to their census tract of residence using ArcGIS, version 10.1 (ESRI).^22^ This study was reviewed and approved by the Washington State Institutional Review Board and PHSKC Research Administration Review Committee. The requirement for patient informed consent was waived by the institutional review board because routinely collected patient records were used in the study, and the study posed no more than minimal risk to patients. This study followed the Strengthening the Reporting of Observational Studies in Epidemiology (STROBE) reporting guideline.^26^

### Study Cohort

The study was conducted within a cohort of children registered in the WA-IIS, which tracks immunization records for people of all ages in Washington state.^23^ Birth certificates of children born in King County are loaded into the registry every 2 weeks. Health care professionals voluntarily report patient immunizations to WA-IIS. The cohort was restricted to children born in King County after January 1, 2008, to ensure data completeness and accuracy. Ninety-nine percent of children aged 4 months to 5 years had at least 2 immunizations recorded in the WA-IIS.^23^ Using WA-IIS data, we created a cohort of 316 404 children aged 3 months to 9 years, born or living in King County, Washington, between January 1, 2008, and December 31, 2017. Vaccination name and date of administration were obtained for all pediatric vaccines recommended from birth to 9 years of age. We assumed that if a child had no record of a DTaP dose in the WA-IIS, they did not receive it. Demographic information included date of birth, sex, current residential address, and county. Home addresses (or zip codes when home addresses were unavailable) of WA-IIS participants were geocoded to their census tract of residence by the Washington Department of Health staff. We calculated a census tract–level Neighborhood Socioeconomic Status score for each participant using the 2010 US census data.^24,25^

### Linking Surveillance and Immunization Data Sets

Immunization records from the WA-IIS and surveillance data from PHSKC were linked based on a probabilistic matching algorithm that used each participant’s first name, last name, date of birth, sex, and city of residence.^27^ Matching was performed using the fastLink package in R, version 3.6.1 (R Core Team)^28^ (eFigure 1 in the Supplement).

### Censoring of Registry Participants

Participants were followed in time until they were diagnosed with pertussis, died, moved to another county, changed health care professionals (entered in the registry as *moved or gone elsewhere*), or until the end of follow-up on December 31, 2017, whichever came first. Vaccination dates for pediatric vaccines were considered proxies for continued enrollment in the WA-IIS and residence in King County. The WA-IIS flags participants as inactive if they move out of state, but this variable was inconsistently recorded. We assumed that participants were active unless they were marked as inactive or died, and the date they became inactive was recorded (eMethods, eFigure 2 in the Supplement).

### Timeliness of DTaP Doses in the Cohort

Age at vaccination was calculated using date of birth and date of vaccination. Days undervaccinated for each DTaP dose were estimated using the Centers for Disease Control and Prevention Advisory Committee on Immunization Practices recommendations for minimum ages of vaccination and minimum acceptable intervals between doses (Table 1)^29^ and a metric described by Luman, et al.^12,13^ Children were considered to be age-appropriately vaccinated without delay if they received each DTaP dose within 4 days before the minimum acceptable age through 30 days after the recommended age range.^29^

**Table 1.** The Advisory Committee on Immunization Practices Recommendations for DTaP Vaccine Schedule and Interval for the US

DTaP dose	Age at administration, mo	Minimum age at administration, d^a^^,^^b^	Interval between doses, d	Age of child after which delay count starts, d
1	2	38		90
2	4	66	28	150
3	6	94	28	210
4	15-18	266	180	578
5^c^	48-84	1456	180	2555

Abbreviation: DTaP, diphtheria-tetanus–acellular pertussis.

^a^ Grace period of 4 days included in the minimum age of administration.

^b^ Weeks and months are converted to days based on the method in Luman et al.^12^

^c^ No dose 5 required if dose 4 is given after 4 years of age.

Children who received fewer than recommended doses at a given age (<3 doses by age 19 months, <4 doses by age 5 years, and <5 doses through age 9 years) were defined as undervaccinated. Children who received the recommended number of doses by a given age but received them outside the recommended window specified in Table 1 were defined as delayed. For example, a child who received DTaP dose 3 at age 9 months instead of 6 months was considered delayed but not undervaccinated at age 19 months.

### Statistical Analysis

Trends in vaccine timeliness by birth cohort were analyzed using Poisson regression. Age-appropriate DTaP uptake over time was estimated by the Kaplan-Meier method with age as the timescale.

We measured association between timeliness of DTaP primary series, second-year booster, and preschool booster with pertussis risk through ages 19 months, 5 years, and 9 years, respectively. The periods of follow-up were selected based on the ages through which the previous doses were expected to provide protection before the next dose is due. Even though dose 5 is recommended between ages 4 and 6 years, we used a cutoff of age 5 years because most children start preschool by this age and begin to interact more with children from other households. We also measured the association between delay in series initiation and pertussis risk through 12 months of age. Between countries, there is considerable variation in DTaP primary series schedules. For instance, Scandinavian countries recommend the “2p + 1” or long series (doses at 2, 4, and 11-12 months), rather than a “3p” or accelerated series (doses at 2, 4, and 6 months).^4,11^ To examine whether difference in vaccine schedules was associated with pertussis risk in infants, we compared pertussis risk between children who happened to receive a 3p schedule vs a 2p + 1 schedule in our cohort.

For association between timeliness of DTaP primary series and pertussis risk, children were followed from ages 7 to 19 months or to pertussis diagnosis or censoring. For vaccine delay only, we restricted the cohort to children who received 3 DTaP doses by age 19 months.

Similarly, for association between undervaccination for second-year booster and pertussis risk, children were followed from ages 19 to 60 months or pertussis diagnosis or censoring. For vaccine delay only, we restricted the cohort to children who received 4 or more DTaP doses by age 60 months. Finally, for the preschool booster, follow-up began at age 60 months and continued through age 9 years or until pertussis diagnosis or censoring. For association between delayed preschool booster and pertussis risk, we restricted the cohort to children who received 5 or more DTaP doses.

For association between delay in series initiation and pertussis risk in the first year of life, children were monitored from ages 3 to 12 months. For comparing accelerated and long schedules, follow-up was from ages 3 to 24 months.

Log-binomial models were used to estimate pertussis risk ratios comparing children with delayed or missing vaccinations to those with timely vaccinations. Person-time at risk in months was used as an offset. Only patients diagnosed during the follow-up period were included in the respective models. Patients diagnosed before the start of the follow-up period were excluded. Children diagnosed with pertussis after the end of the follow-up period contributed time at risk in each model. All models were adjusted for NSES score and age. Significance was defined as *P* < .05, and all hypothesis tests were 2-sided. Data were analyzed from June 30 to December 1, 2019. Data analyses were done using R, version 3.6.1 (R Core Team).^30^

## Results

The analyses included 316 404 children aged 3 months to 9 years (162 025 boys [51.2%] and 154 379 girls [48.8%]) who contributed 17.4 million person-months of follow-up. The median age as of December 31, 2017, was 65.2 months (interquartile range, 35.3-94.1 years). A total of 19 943 children (6.3%) had no DTaP dose recorded in the WA-IIS, 116 193 (36.7%) were delayed for at least 1 DTaP dose, and 180 268 (56.9%) were fully vaccinated with no delay (Table 2). A higher proportion of both unvaccinated children (4368 [21.9%] vs 3610 [18.1%]) and children who received delayed vaccination (28 487 [24.5%] vs 18 799 [16.2%]) resided in census tracts with the lowest NSES quintile (Q1) compared with the highest NSES quintile (Q5). Of the 404 of 438 pertussis cases (92%) that were successfully linked to WA-IIS participants (eFigure 1 in the Supplement), 116 children (28.7%) were unvaccinated, 149 (36.9%) were delayed, and 139 (34.4%) received on-time and age-appropriate vaccinations. A total of 111 children (26%) were aged 6 months or younger, 54 (10%) were aged 7 to 11 months, 208 (47.5%) were aged 1 to 4 years, and 65 (13.6%) were aged 5 to 9 years. Forty-nine children (11.2%) required hospitalization.

**Table 2.** Characteristics of Washington State Immunization Information System Participants Aged 3 Months or Older Born or Living in King County, Washington, Between 2008 and 2017 by Diphtheria-Tetanus–Acellular Pertussis Vaccination Status

Characteristic	No. (%)		
	Unvaccinated^a^ (n = 19 943)	Delayed^b^ (n = 116 193)	Not delayed (n = 180 268)
Sex			
Male	10 165 (50.9)	59 374 (51.1)	92 486 (51.3)
Female	9778 (49.0)	56 819 (48.9)	87 782 (48.7)
NSES score^c^			
Q1 (lowest)	4368 (21.9)	28 487 (24.5)	31 997 (17.7)
Q2	3909 (19.6)	24 727 (21.3)	32 823 (18.2)
Q3	4077 (20.4)	23 271 (20.0)	36 506 (20.2)
Q4	3884 (19.5)	20 589 (17.7)	37 524 (20.8)
Q5 (highest)	3610 (18.1)	18 799 (16.2)	40 817 (22.6)
Pertussis cases	116 (28.7)	149 (36.9)	139 (34.4)
Median age of follow-up, mo	11.8	61.9	49.2
Birth cohort			
2008	2154 (10.8)	15 386 (13.2)	17 992 (9.9)
2009	1991 (9.9)	14 472 (12.4)	18 194 (10.1)
2010	1813 (9.1)	13 824 (11.9)	18 085 (10.0)
2011	1723 (8.6)	13 149 (11.3)	18 403 (10.2)
2012	1468 (7.4)	12 932 (11.1)	18 825 (10.4)
2013	1761 (8.8)	12 599 (10.8)	17 721 (9.8)
2014	2192 (10.9)	11 083 (9.5)	18 515 (10.3)
2015	2310 (11.6)	9959 (8.6)	18 422 (10.2)
2016	2492 (12.5)	8383 (7.2)	19 160 (10.6)
2017	2039 (10.2)	4406 (3.8)	14 951 (8.3)

Abbreviation: NSES, Neighborhood Socioeconomic Status.

^a^ Unvaccinated children have 0 doses of diphtheria-tetanus–acellular pertussis vaccine recorded in the Washington Immunization Information System.

^b^ At least 1 diphtheria-tetanus–acellular pertussis dose that is delayed.

^c^ The Neighborhood Socioeconomic Status score was divided into quintiles Q1-Q5, where Q1 is the quintile with the lowest Neighborhood Socioeconomic Status score (20th percentile or lower) and Q5 is the quintile with the highest Neighborhood Socioeconomic Status score (80th percentile or higher) scores.

Delayed vaccination and undervaccination was higher for older ages (17.6% delayed or undervaccinated for dose 1 at age 3 months vs 41.6% at age 5 years for dose 5) (eTable 1 in the Supplement), but vaccine delay among children who eventually received the doses was not longer than 5 weeks. Timeliness improved for successive birth cohorts (52.2% for 2008 birth cohort vs 32.3% for 2017 birth cohort; yearly decrease of delay for dose 1, β = –0.03, SD = 0.03; yearly decrease of delay for dose 5, β = –0.04, SD = 0.05) (eTable 2 in the Supplement). By age 7 years, 86.2% of the cohort was age-appropriately vaccinated (eFigure 3 in the Supplement).

Among children aged 7 to 19 months, adjusted relative risk (aRR) of pertussis was 4.8 times higher (95% CI, 3.1-7.6) for children undervaccinated or delayed for the primary series, compared with those who received age-appropriate and timely vaccination. When restricted to children with 3 doses or more, this association was not statistically significant (aRR, 0.8; 95% CI, 0.3-2.2). For children aged 19 to 60 months, risk of pertussis was 3.2 times higher (95% CI, 2.3-4.5) among children who were undervaccinated or delayed for the second-year booster. For children aged 5 to 9 years, risk of pertussis was 4.6 times higher (95% CI, 2.6-8.2) among children who were undervaccinated or delayed for the preschool booster. Again, delay in booster doses was not associated with elevated pertussis risk when an age-appropriate number of doses were given (Table 3).

**Table 3.** Estimated Relative Risk of Pertussis Comparing Children Who Were Undervaccinated or Vaccinated With Delay With Those Who Received Age-Appropriate and Timely DTaP Vaccine Using Log-Binomial Models

Model	Cohort	Cohort size	Start follow-up time, mo	End follow-up time, mo	PT at risk in exposed, mo	PT at risk in unexposed, mo	Cases exposed^**a**^	Cases unexposed^**b**^	aRR (95% CI)^**c**^
**Primary series**										
Undervaccinated with or without delay vs age-appropriate and timely vaccination	Children ≥7 mo	298 166	7	19	949 469.4	2 671 653	54	31	4.8 (3.1-7.6)
Delayed vs timely doses among those with age-appropriate vaccination	Children ≥7 mo who received ≥3 doses	257 913	7	19	492 272.2	2 671 653	5	31	0.8 (0.3-2.2)
**First booster**										
Undervaccinated with or without delay vs age-appropriate and timely vaccination	Children ≥19 mo	258 675	19	60	2 598 849	5 360 429	99	59	3.2 (2.3-4.5)
Delayed vs timely doses among those with age-appropriate vaccination	Children ≥19 mo who received ≥4 doses	221 928	19	60	1 732 007	5 360 429	17	59	0.8 (0.5-1.4)
**Second booster**										
Undervaccinated with or without delay vs age-appropriate and timely vaccination	Children ≥60 mo	134 950	60	Age censored /end of study	1 327 522	2 664 899	38	17	4.6 (2.6-8.2)
Delayed vs timely doses among age-appropriately vaccinated	Children ≥60 mo or older who received ≥5 doses	111 387	60	Age censored/end of study	580 387.7	2 664 899	5	17	1.3 (0.5-3.6)
**Series initiation**										
Undervaccinated with or without delay dose 1 vs age-appropriate and timely vaccination for primary series	Children ≥3 mo	301 494	3	12	579 975.3	3 006 319	38	54	3.5 (2.3-5.5)
Delayed vs timely dose 1 among age-appropriately vaccinated for primary series	Children ≥3 mo who received 3 doses by 7 mo of age	295 325	3	12	64 926.5	2 555 811	0	41	NA
3p vs 2p + 1 schedule^d^	Children ≥3 mo	301 494	3	24	4 870 512	307 677.8	1	60	3.9 (0.5-28.8)

Abbreviations: aRR, adjusted relative risk; DTaP, diphtheria-tetanus–acellular pertussis; PT, person-time.

^a^ Exposed group: undervaccination with or without vaccination delay for models assessing effect of undervaccination on pertussis risk and vaccination delay for models assessing effect of vaccination delay only on pertussis risk.

^b^ Unexposed group: age-appropriate and timely vaccination with DTaP vaccine.

^c^ Adjusted relative risk are risk ratios adjusted for age and Neighborhood Socioeconomic Status score.

^d^ The term “3p” indicates accelerated 3-dose DTaP primary series to be administered at ages 2, 4, and 6 months; “2p + 1” indicates long 3-dose DTaP primary series to be administered at ages 2, 4, and 11/12 months; 2p + 1 is the exposed group.

Among those who received 3 DTaP doses by age 7 months, delay in series initiation was not associated with pertussis risk. However, those who initiated the series late were also 48% less likely (95% CI, 47%-49%) to complete the primary series, and undervaccination with the primary series was associated with 3.5-fold higher (95% CI, 2.3-5.5) pertussis risk in the first year of life. There was no difference in pertussis risk between children who received the 3p vs 2p + 1 schedule (Table 3).

## Discussion

In this cohort study, we measured the association between both vaccine timeliness and number of doses with age-specific pertussis risk among infants and young children in King County, Washington. We found that receiving fewer than the recommended number of doses by a given age was associated with higher pertussis risk in children, despite high overall vaccine coverage in King County. Even when administered with short delays, getting the primary series in the first year of life and the 2 booster doses in the second and fifth year of life was associated with lower childhood pertussis risk. However, deliberately delaying doses is not recommended, because those who delayed were less likely to finish the series.

Linking the WA-IIS immunization data with pertussis surveillance data from PHSKC allowed us to create a population-based cohort with near-complete ascertainment of pertussis vaccination status for more than 315 000 children aged 0 to 9 years. The WA-IIS has a high degree of internal and external validity, and the vaccination and demographic data elements are highly complete, making it a useful tool for answering our research question.^31^ This data set also allowed us to directly compare the long and accelerated primary schedules within the same population, which has been otherwise challenging owing to the underlying difference in pertussis epidemiology between countries.^32^

Other studies have found vaccine delay to be associated with higher pertussis risk but did not differentiate between children who received fewer than the recommended number of doses and those who received all the doses but with a delay.^14,16,33^ Additional doses, even when delayed, can give more protection against pertussis, so differentiating between delayed vaccination and undervaccination is worthwhile. These studies also did not measure the association between undervaccination and pertussis for the appropriate age groups at risk, potentially resulting in misclassification of person-time at risk. For example, Huang et al^14^ measured the association of delay in any of the 4 of DTaP vaccine doses with pertussis incidence among all children aged 3 to 36 months compared with no delay. Person-time at risk was carefully assigned in our study and our findings supported the Advisory Committee on Immunization Practices recommendations for vaccine schedules in the US.

Despite the World Health Organization’s recommendation that the 3-dose DTaP primary series should be completed by 6 months of age for enhanced infant protection,^4,34^ we found no difference in pertussis risk between those who received the 3p vs 2p +1 schedule. This might be due to the small sample size (<6%) of cohort members who received the 2p +1 schedule. A systematic review comparing effectiveness of different immunization schedules against pertussis drew similar conclusions.^11^ Even so, countries such Finland, Norway, and Sweden, which use the 2p +1 schedule, have not experienced a resurgence in pertussis, unlike the US, which uses the 3p schedule. Potential reasons might be that vaccine coverage in these countries is very high during infancy and preschool age and that a booster dose at 12 months might provide better protection after the first year.^4,11^

Similar to a study in Netherlands, we found that initiating the series earlier than 3 months of age may not be as important as receiving all recommended vaccine doses in the series within the first 6 months of life.^35^ However, children who delayed the primary series were also less likely to complete it in the first year of life, confirming findings of a study done in Australia.^36^ There is evidence for incremental protection after each additional dose, so being completely vaccinated with 3 primary doses is essential for full protection against pertussis.^8,37^ Thus, clinicians should encourage parents to initiate DTaP primary series at the earliest recommended age to ensure series completion and protection against pertussis.

Our study results support the World Health Organization’s recommendation of a second-year booster at age 18 months. Pertussis incidence was higher among children aged 2 to 5 years who had fewer than 4 doses in their second year of life. Australia experienced a similar increase in pertussis incidence among children aged 2 to 3 years when they discontinued the 18-month booster in 2003; this policy change is considered to be one of the drivers of pertussis resurgence in Australia.^38^ Similarly, those who received 5 or more doses by 7 years of age had lower risk of pertussis through age 9 years, providing support for the recommendation of a preschool booster in the US. Modeling studies have shown that, at least in the US, school-aged children are core transmission groups that help sustain pertussis transmission chains owing to increased contact rates.^39,40^ Thus, a booster dose given to school-aged children between 4 and 6 years of age could be crucial to protect them against pertussis as well as to reduce overall pertussis transmission.

### Limitations

Our study had some limitations. First, because our case definition was highly specific with strict clinical diagnosis criteria, cases in our study may have been more severe and likely to have been underreported when compared with cases in studies that relied on more sensitive but less specific polymerase chain reaction diagnostic techniques for case detection.^41^ Second, our estimates could be biased owing to measurement errors. If the 34 cases that were excluded because they could not be linked to the WA-IIS were also likely to represent undervaccinated children, then the current estimated association between undervaccination and pertussis risk is an underestimate. Pertussis cases may have been misclassified as undervaccinated because health care professionals failed to report doses to the WA-IIS, which may have resulted in overestimation of the association between undervaccination and pertussis risk. If children had shorter or longer follow-up time than what we assigned by our censoring algorithm, relative risks could be biased in either direction. Third, the WA-IIS does not capture important confounders such as household size, adolescent siblings, maternal education, and day care or school attendance. Fourth, the results of this study are generalizable only to countries that administer acellular pertussis vaccines and use the same schedule as the US.

## Conclusions

In this cohort study, undervaccination with DTaP vaccine was associated with a higher pertussis risk for infants and young children. The current 5-dose DTaP vaccine series recommended by the Advisory Committee on Immunization Practices and the World Health Organization protects against childhood pertussis. Given this study’s findings, policies should emphasize receiving all doses of pertussis vaccine at the recommended ages, even if there is a short delay. Parents should be encouraged to follow recommended vaccine schedules, and in the event of a delay, the next dose in the series should be administered at the earliest possible opportunity.

## Notes

Supplement. **eFigure 1.** Flowchart Describing Probabilistic Matching of Surveillance Database (Pertussis Cases) With Immunization Registry (Immunizations) **eMethods.** Censoring of Cohort Participants **eFigure 2. **Flowchart Describing Censoring Algorithm **eTable 1.** Timeliness of DTaP Vaccine by Dose Among Washington State Immunization Information System Participants Born or Living in King County, WA, Between 2008-2017 **eTable 2. **Trends in Vaccination Delay for DTaP Doses From 2008 to 2017 Estimated by Poisson Regression **eFigure 3. **Age-Appropriate DTaP Vaccination Using Inverse Kaplan-Meier Curves for 0 to <10 Year Olds in King County, WA
eReferences
