# Supplementary material for: Association of Diphtheria-Tetanus–Acellular Pertussis Vaccine Timeliness and Number of Doses With Age-Specific Pertussis Risk in Infants and Young Children
Source: JAMA Netw Open. 2021 Aug 10;4(8):e2119118. doi: 10.1001/jamanetworkopen.2021.19118 (PMC8356064; doi:10.1001/jamanetworkopen.2021.19118)
Supplement: Supplement. — eFigure 1. Flowchart Describing Probabilistic Matching of Surveillance Database (Pertussis Cases) With Immunization Registry (Immunizations) eMethods. Censoring of Cohort Participants eFigure 2. Flowchart Describing Censoring Algorithm eTable 1. Timeliness of DTaP Vaccine by Dose Among Washington State Immunization Information System Participants Born or Living in King County, WA, Between 2008-2017 eTable 2. Trends in Vaccination Delay for DTaP Doses From 2008 to 2017 Estimated by Poisson Regression eFigure 3. Age-Appropriate DTaP Vaccination Using Inverse Kaplan-Meier Curves for 0 to <10 Year Olds in King County, WA eReferences [file jamanetwopen-e2119118-s001.pdf]

# Supplementary Online Content

Rane MS, Rohani P, Halloran ME. Association of diphtheria-tetanus–acellular pertussis vaccine timeliness and number of doses with age-specific pertussis risk in infants and young children. *JAMA Netw Open*. 2021;4(8):e2119118. doi:10.1001/jamanetworkopen.2021.19118

**eFigure 1.** Flowchart Describing Probabilistic Matching of Surveillance Database (Pertussis Cases) With Immunization Registry (Immunizations)

**eMethods.** Censoring of Cohort Participants

**eFigure 2.** Flowchart Describing Censoring Algorithm

**eTable 1.** Timeliness of DTaP Vaccine by Dose Among Washington State Immunization Information System Participants Born or Living in King County, WA, Between 2008-2017

**eTable 2.** Trends in Vaccination Delay for DTaP Doses From 2008 to 2017 Estimated by Poisson Regression

**eFigure 3.** Age-Appropriate DTaP Vaccination Using Inverse Kaplan-Meier Curves for 0 to <10 Year Olds in King County, WA

**eReferences**

This supplementary material has been provided by the authors to give readers additional information about their work.

© 2021 Rane MS et al. *JAMA Network Open.*

**eFigure 1.** Flowchart Describing Probabilistic Matching of Surveillance Database (Pertussis Cases) With Immunization Registry (Immunizations)

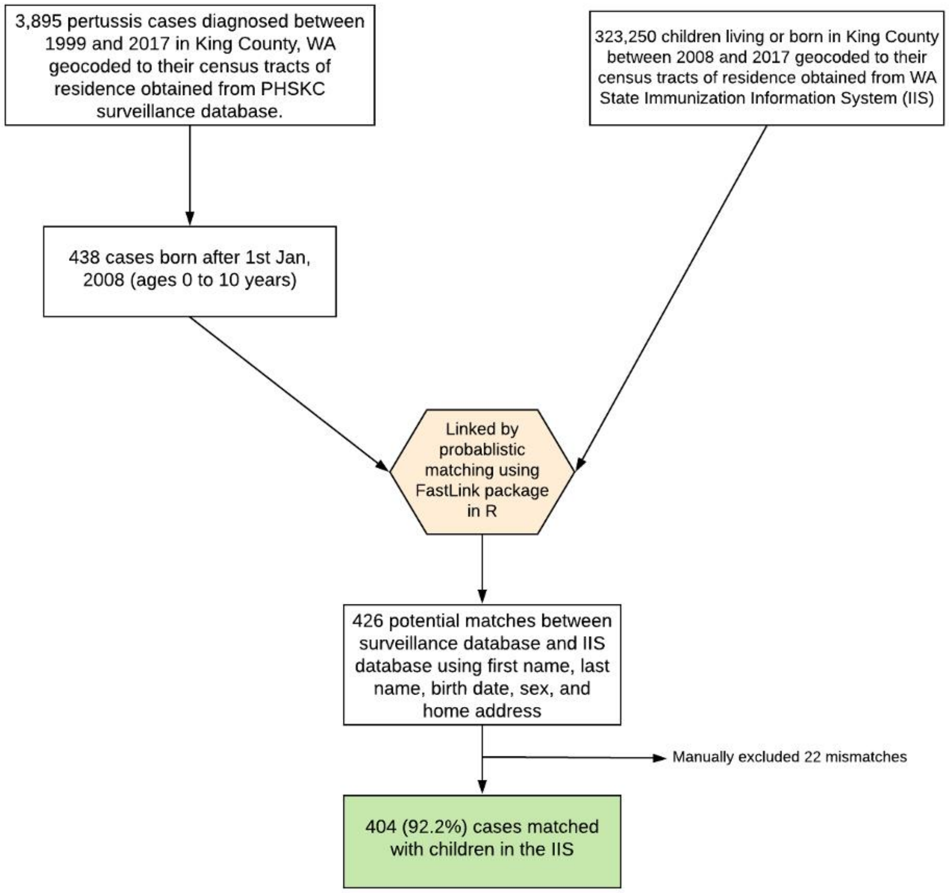


Immunization records from the WA-IIS and surveillance data from PHSKC were merged based on a probabilistic matching algorithm that used participants' first name, last name, date of birth, sex, and city of residence [1]. Matching was performed using the fastLink package in R [2]. First and last names were linked using string distance matching and partial matches were allowed for these fields. All pairs that were matched with a posterior probability of > 0.85 were retained for further review and false matches were removed manually. Unmatched recorded were reviewed and matched manually based on additional data in the surveillance and immunization registry. After matching was completed, all personal identifiers were removed.

**eMethods.** Censoring of Cohort Participants

The following rules were used sequentially for censoring individuals in this study:

i. Participants diagnosed with pertussis were censored on the date of diagnosis.

ii. If no immunization (any pediatric immunization) is ever recorded for a participant, then they are censored on their first birthday.

iii. For participants indicated as “active" in the WA-IIS and whose current residence is King County, follow-up ended at two years after the last recorded vaccine if last vaccine was recorded at age < 36 months, or at the end of study period if last vaccine was recorded at age ≥36 months.

iv. For active and inactive participants that no longer live in King County, follow-up ended on the date they moved out of King County or changed provider (recorded as Date MOGE or “Moved Or Gone Elsewhere" in the WA-IIS), if this date was recorded.

v. For inactive participants missing MOGE date, follow up ended on the date their record was last updated for any reason (recorded as Date Last Update in the WA-IIS), assuming that they were King County residents at least until that date.

Length of follow up was assigned based on ages of last recorded vaccines, under the assumption that the WA-IIS will have a complete immunization record for children who continue to reside in King County. We assigned the shortest time of follow up to those with no immunizations in the registry (1 year). Almost all pediatric vaccination series start before the age of 36 months and boosters are administered after 36 months. Thus, we assigned a shorter follow up time (2 years after last vaccine recorded) to those who did not have any doses recorded after 36 months of age. For the rest, we assumed that follow up ended at 9 years of age (or end of study follow up time), in the absence of additional information indicating they are no longer part of WA-IIS.

© 2021 Rane MS et al. *JAMA Network Open.*

**eFigure 2.** Flowchart Describing Censoring Algorithm

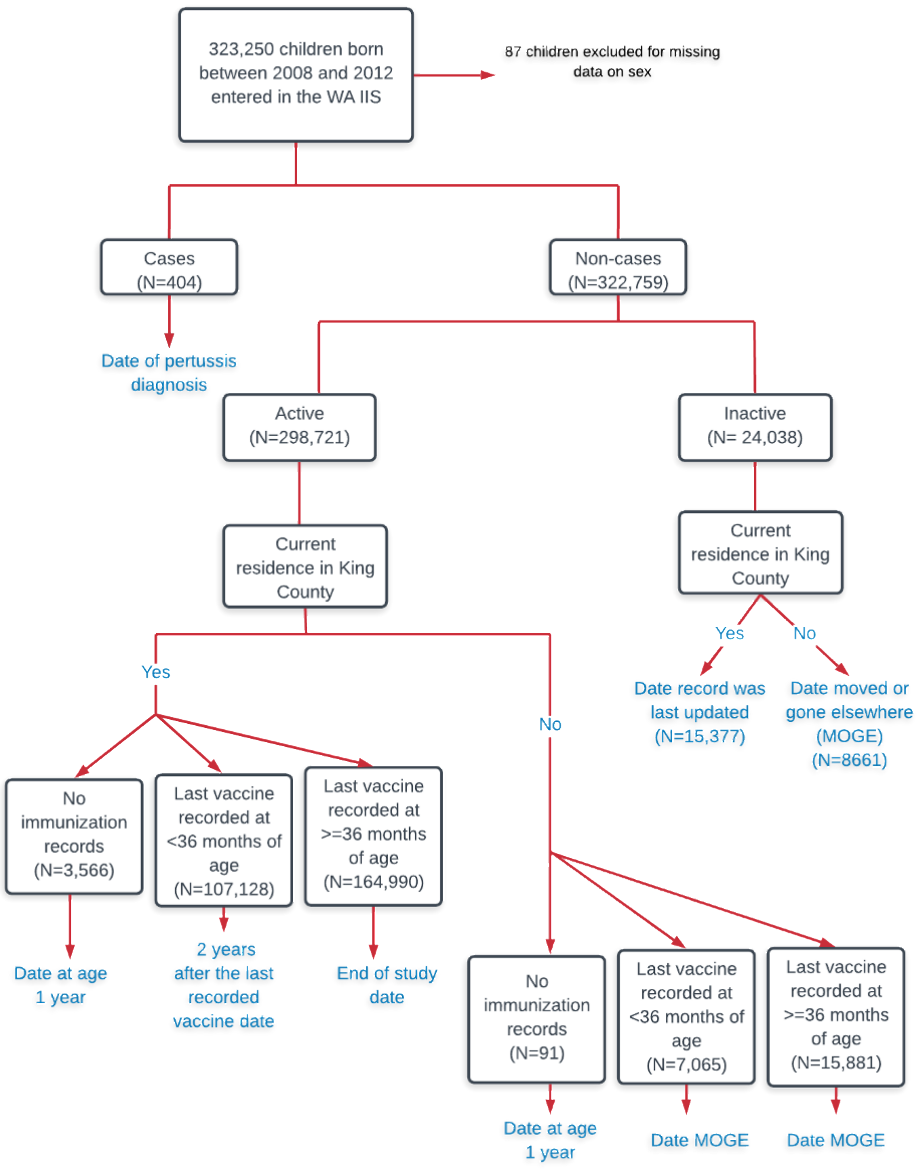


All pediatric vaccines, except Influenza vaccines, administered to the children registered in the WA-IIS and recorded by the WA-IIS were used to determine censoring. Vaccines of all formulations (including unspecified formulations) and combinations against Hepatitis A, Hepatitis B, Haemophilus Influenza B, Poliomyelitis, Diphtheria-Tetanus-acellular-Pertussis, Meningococcus, Measles-Mumps-Rubella, Varicella, Pneumcoccus, Rotavirus were used. Historical shots for any of the recorded in the registry were also used.

© 2021 Rane MS et al. *JAMA Network Open.*

**eTable 1.** Timeliness of DTaP Vaccine by Dose Among Washington State Immunization Information System Participants Born or Living in King County, WA, Between 2008-2017

| Vaccine dose | Measured at age | Timeliness | | | | | |
|---|---|---|---|---|---|---|---|
| | | On-time, N(%) | Delayed, N(%) | Never, N(%) | Total | Mean age (months) | Mean delay (days) |
| Dose 1 | 3 months | 260707 (82.4) | 35754 (11.3) | 19943 (6.3) | 316404 | 3.46 | 13.8 |
| Dose 2 | 5 months | 232837 (74.71) | 50727 (16.28) | 28096 (9.01) | 311660 | 5.53 | 16.1 |
| Dose 3 | 7 months | 204534 (66.65) | 67895 (22.13) | 34435 (11.22) | 306864 | 8.24 | 37.3 |
| Primary series | 7 months | 219799 (71.6) | 52630 (17.2) | 34435 (11.2) | 306864 | 8.24 | |
| Booster 1 | 19 months | 175113 (63.14) | 56518 (20.38) | 45696 (16.48) | 277327 | 20.42 | 34.6 |
| Booster2 | 60 months | 99614 (58.46) | 50388 (29.57) | 20409 (11.98) | 170411 | 52.88 | -123 |

eTable1 describes timeliness of DTaP doses in this cohort. Delay in vaccination and under-vaccination increased with age. A total of 19,943 (6.3%), 28,096 (9%), and 34,435 (11.2%) of children eligible for DTaP doses 1, 2 and 3 respectively, did not receive it. Of the ones that did receive primary doses, 35,754 (11.3%) were delayed for dose 1 (mean delay 13.8 days), 50,727 (16.3%) were delayed for dose 2 (mean delay 16.1 days), and 67,895 (22.1%) were delayed for dose 3 (mean delay 37.3 days). Thus, vaccine delay among children who eventually received them was not longer than 5 weeks. Of the children eligible for DTaP boosters, 45,696 (16.5%) never received their second-year booster and 56,518 (20.4%) were delayed, whereas 20,409 (12%) never received their preschool booster and 50,388 (29.6%) were delayed. Again, mean delay for boosters among those who did receive them was not substantial likely due to wider windows of recommended ages.

**eTable 2.** Trends in Vaccination Delay for DTaP Doses From 2008 to 2017 Estimated by Poisson Regression

| DTaP Dose number | Trend in delay, $\beta$ (SE) |
|---|---|
| 1 | -0.03 (0.03) |
| 2 | -0.01 (0.02) |
| 3 | -0.01 (0.02) |
| 4 | -0.02 (0.02) |
| 5 | -0.04 (0.05) |

Trend analysis suggests while timeliness for each dose improved for successive birth cohorts (β is negative), this change was not statistically significant.

© 2021 Rane MS et al. *JAMA Network Open.*

**eFigure 3.** Age-Appropriate DTaP Vaccination Using Inverse Kaplan-Meier Curves for 0 to <10 Year Olds in King County, WA

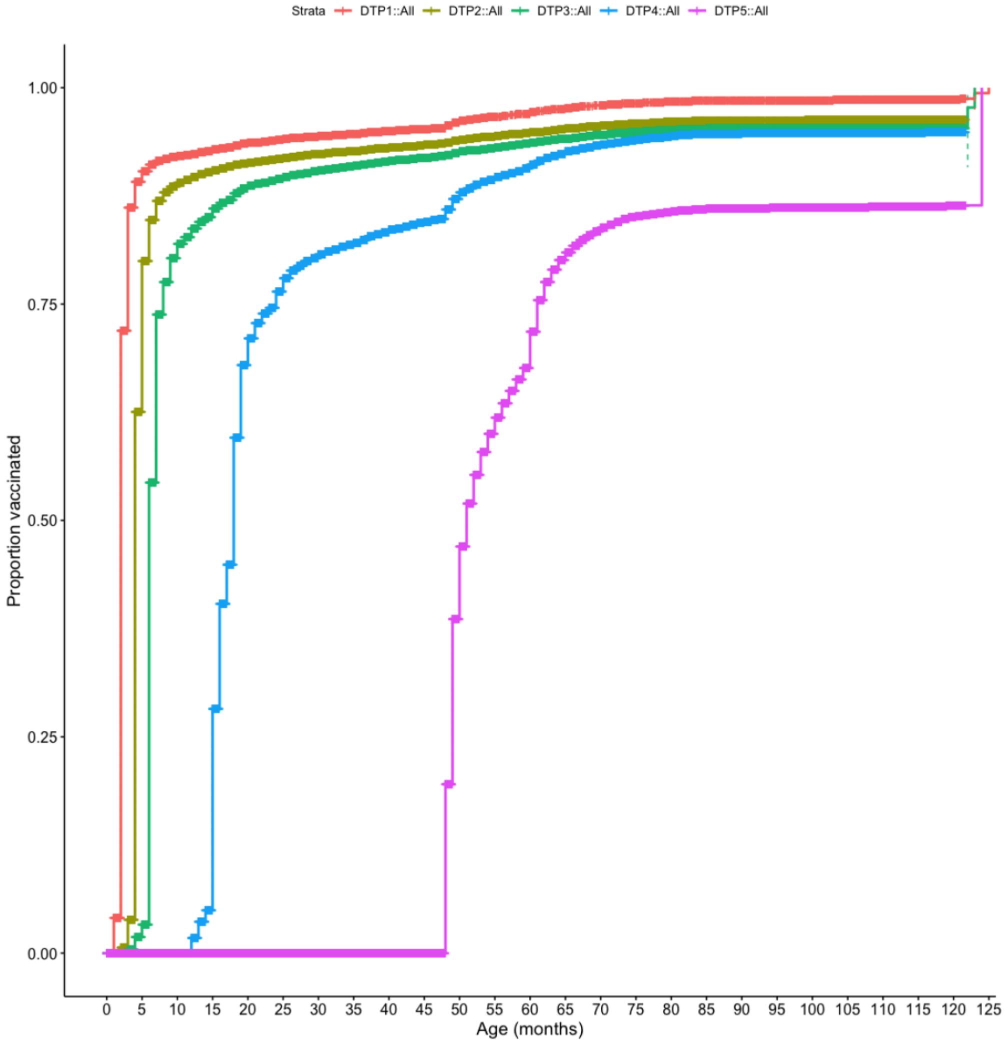


Proportion of 323,163 children aged 0 to 9 years vaccinated with DTaP1 (red), DTaP2 (yellow), DTaP3 (green), DTaP4 (blue), and DTaP5 (purple) at each age and 95% CIs are displayed using inverse Kaplan-Meier curve. Children in this analysis were born between January 2008 and December 2017. The 95% CIs are very narrow because the sample size is very large.

Age-appropriate DTaP uptake over time was estimated by the Kaplan-Meier method with age as the timescale and is plotted in eFigure3. Vaccination coverage at age *a* (in months) was estimated by 1-S(*a*), where the Kaplan-Meier survival function S(*a*) is the cumulative probability of being vaccinated by age *a*.

By 3 months of age, 86.2% (95% CI: 86.1%, 86.3%) of the cohort had received DTaP dose 1. By 5 months of age, 80% (95% CI: 79.9%, 80.2%) of the cohort had received DTaP dose 2. By 7 months of age, 73.9% (95% CI: 73.7%, 74.0%) had received DTaP dose 3, while > 90% coverage with three doses, which is nationally recommended, was achieved by age 14 months [3]. By 19 months of age, 67.9% (95% CI: 67.7%, 68.1%) had received DTaP4 and > 85% coverage for DTaP dose 4 was achieved by 48 months of age. By age 7 years, 86.2% (95% CI: 85.8%, 86.1%) of cohort had received DTaP dose 5, meeting the national vaccine coverage target for five doses of DTaP

© 2021 Rane MS et al. *JAMA Network Open.*

**eReferences**


1. Sayers A, Ben-Shlomo Y, Blom AW, Steele F. Probabilistic record linkage. Int J Epidemiol **2016**; 45:954–964.
2. Enamorado T, Fifield B, Imai K. Fast Probabilistic Record Linkage with Missing Data. 2018;
3. U.S. Department of Health and Human Services. Office of Disease Prevention and Health Promotion. Washington DC. Healthy People 2020. Available at: https://www.healthypeople.gov/2020/topics-objectives/topic/immunization-and-infectious-diseases/objectives. Accessed 3 June 2020.


© 2021 Rane MS et al. *JAMA Network Open.*
